# Strengthening health information systems and inherent statistical outputs for improved malaria control and interventions in western Kenya

**DOI:** 10.3389/fepid.2025.1591261

**Published:** 2025-06-19

**Authors:** Taliyah Griffin, Felix Pabon-Rodriguez, George Ayodo, Yan Zhuang

**Affiliations:** ^1^ Department of Biostatistics and Health Data Science, Indiana University School of Medicine, Indianapolis, IN, United States; ^2^ Department of Biomedical Engineering and Informatics, Indiana University Indianapolis, Luddy School of Informatics, Computing, and Engineering, Indianapolis, IN, United States; ^3^ Centre for Community Health and Wellbeing, Jaramogi Oginga University of Science and Technology, Bondo, Kenya

**Keywords:** malaria, statistical analysis, health information system, sub-Saharan Africa, surveillance, Kenya

## Abstract

Malaria control efforts in Kenya face persistent challenges due to fragmented health information systems, despite notable digital innovations. This mini review evaluates implementations in western Kenya, contrasting successes like Siaya County's Electronic Community Health Information System (eCHIS), developed through collaborations between the Ministry of Health, local agencies, and frontline health workers, which reduces reporting delays through real-time mobile data collection, with ongoing struggles including paper-based records in health facilities and unreliable rural internet. We document how analytical methods, when properly supported, can transform surveillance. Methods such as spatiotemporal models using climate and case data can improve outbreak predictions, while machine learning techniques can optimize insecticide-treated bed net distributions by pinpointing high-risk households. However, these analytical tools remain underutilized due to data fragmentation and limited technical capacity. Key implementation challenges emerged, including device charging difficulties for community health workers, inconsistent data standards between systems, and privacy concerns under Kenya's new Digital Health Act that policymakers are currently addressing through revised guidelines. Key recommendations from this review include the expansion of digital health platforms with co-design input from end-users, improved data quality through standardized reporting mechanisms enforced by county health leadership, and the incorporation of predictive modeling to identify high-risk areas and optimize intervention timing. Investing in robust health information infrastructure will not only strengthen malaria control efforts in Kenya but also serve as a model for other malaria-endemic regions. Digital tools show tremendous potential when paired with sustained training, community engagement, and realistic maintenance solutions supported by public-private partnerships.

## 1 Introduction

Malaria is a life-threatening disease caused by *Plasmodium* parasites, transmitted to humans through the bites of infected *Anopheles* mosquitoes (1, 2). Despite being preventable and treatable, malaria continues to be a major public health burden, particularly in sub-Saharan Africa, with an estimated 263 million cases and 597,000 malaria deaths worldwide in 2023 with an increasing incidence rate from 58.6 in 2022 to 60.4 cases per 1,000 population at risk in 2023 (2). An estimated 95% of these cases are from the World Health Organization (WHO) African Region with a mortality rate of 13.7 per 1,000 population (2). However, in Kenya, approximately, 3.4 million malaria cases and over 12,000 deaths have been reported (3). Due to the scale-up of interventions which include insecticide-treated nets (ITNs), indoor spraying (IRS), and Artemisinin-based combination therapy, morbidity and mortality have reduced by 27% and 59% in sub-Saharan Africa between 2000 and 2020 (1, 3). Despite the reduction, the prevalence of malaria is still high in the region significantly hampering economic growth and development, particularly in malaria-endemic areas where recurrent infections limit productivity, increase healthcare costs, and weaken health systems (2).

Kenya is among the high-burden malaria countries in Africa, with transmission varying by region due to ecological and climatic factors (4, 5). The western region, particularly Siaya County, experiences some of the highest malaria prevalence rates in the country. Our recent study and surveys indicate that Siaya County has a shift of malaria burden and a malaria prevalence of over 30%, nearly double that of other endemic regions in Kenya (5–7). Several factors contribute to this high burden, including favorable environmental conditions for mosquito breeding, limited access to healthcare, and high rates of asymptomatic infections among school-aged children, which sustain ongoing transmission (4). Effective malaria control in Siaya County requires not only robust intervention strategies but also reliable data systems to track cases, monitor shift of malaria burden or increase of cases, and optimize resource allocation (8). Health information systems (HIS) play a critical role in strengthening disease surveillance, monitoring intervention effectiveness, and supporting data-driven decision-making. In many countries, digital health platforms have transformed disease management. For example, the use of mobile health (mHealth) applications for malaria surveillance has enhanced data collection, and communication in rural areas (9). However, in Kenya, the health information infrastructure remains fragmented, with multiple data systems operating in parallel, the data captured at point-of-care in the community and health facilities are mostly paper-based leading to delays in reporting, inconsistencies in data quality, and limited integration across platforms (10–12). Some major challenges are the lack of standardization in data formats and reporting protocols, making it difficult to merge datasets from different health facilities, community health workers, and research institutions (13–18). Additionally, infrastructure limitations such as unreliable internet access, insufficient digital tools, and gaps in training among healthcare workers further complicate data integration efforts (13, 14, 17, 18). These are among the barriers that hinder real-time malaria tracking, delaying responses to outbreaks and limiting the effectiveness of intervention programs.

The Climate Change and Epidemics 2024 Report by the CLIMADE Consortium highlights several urgent calls for action (19). One key priority is timely outbreak reporting, emphasizing the need for governments and health organizations to commit to immediate and transparent reporting of infectious disease outbreaks, ensuring open data sharing to enhance global preparedness and response. Another critical focus is prioritizing vulnerable populations, recognizing that climate change and infectious diseases disproportionately impact these communities. The report calls for investments in resilient healthcare systems, infrastructure, and disaster preparedness to protect those most at risk. Similarly, the WHO World Malaria Report 2024 emphasizes the need to address inequities in the global malaria response, advocating for better data systems and knowledge to improve surveillance, decision-making, and health outcomes (2).

This review aims to provide a comprehensive overview of the existing health information systems used for malaria surveillance in Kenya, with a specific focus on malaria hotspot area such as Siaya County. It will examine the statistical analyses currently applied within these systems, assess their strengths and limitations, and explore opportunities for improvement. By identifying gaps in data collection, reporting, and integration, this paper seeks to inform policymakers, researchers, and public health officials about potential strategies to enhance malaria surveillance and decision-making. Strengthening health information systems and employing advanced statistical methods will be critical in optimizing malaria control strategies and ultimately reducing the burden of the disease in Kenya and beyond.

## 2 Situation in Western Kenya

### 2.1 Literature search

While this study consist of a short review, we completed a structured search strategy to ensure a comprehensive review of existing health information systems and statistical approaches for malaria control in Western Kenya. We searched multiple academic databases, including PubMed, Web of Science, Scopus, and Google Scholar, using keywords such as “malaria surveillance AND health information systems,” “digital health platforms AND malaria reporting,” “predictive modeling AND malaria transmission,” and “statistical models AND malaria forecasting.” We consider the following studies: (1) peer-reviewed articles, reports, and policy documents published within the last 15 years, (2) studies focusing on malaria surveillance, predictive modeling, and statistical analyses, (3) research conducted in Kenya or similar endemic regions in sub-Saharan Africa, and (4) studies examining health information systems and digital health technologies. We also considered grey literature, such as reports from the World Health Organization (WHO), Kenya Ministry of Health (MOH), Kenya Medical Research Institute (KEMRI), and the CLIMADE Consortium, to incorporate policy-related insights and government initiatives.

### 2.2 Health information systems

The District Health Information Software 2 (DHIS2) is a free, open-source, web-based platform designed for the collection, validation, analysis, and presentation of aggregate and patient-based statistical data across multiple health programs (20). With its flexible metadata model and configurable user interface, DHIS2 allows countries to tailor their health information systems without custom programming. In Kenya, the Kenyan Health Information System (KHIS) is built on DHIS2, transforming fragmented, paper-based reporting into a unified electronic system (21). The KHIS allows for routine reporting of various diseases, including malaria, with individual counties being directly responsible for their own data collection, analysis, and dissemination (22). With this decentralized system, there have been multiple digital health systems aimed at strengthening data collection. However, they have been described as “uncoordinated, fragmented, and not integrated into a cohesive national health framework” (10). For instance, the HIS suffers from documented fragmentation: (1) not all systems are capable of KHIS integration; (2) manually transcribe paper records into digital systems causes delays; and (3) absent of auto synchronization force health workers to spend surveillance time reconciling duplicate entries. These health systems do not “facilitate the integration of different sources of health information within the health system”, ultimately meaning that healthcare providers are unable to share health-related information effectively and on time (23). The lack of interoperability impacts the quality of patient care, the effectiveness of care, and the usability of the data collected (19). This not only affects providers but can complicate data analysis and reporting. At present, the Kenya Health Information System (KHIS) operates as an electronic system that aggregates routine health data from health facilities across the country. However, the method of data capture at the facility and community levels varies. In many facilities, particularly in rural and under-resourced areas, data are initially recorded on paper before being digitized and uploaded into KHIS.

In 2020, the Kenyan government started developing a new program to address the issues previously discussed. The Electronic Community Health Information System (eCHIS), nationally launched in September 2023, has been referred to as the first of its kind in Kenya. The eCHIS follows a “standardized requirements framework,” ultimately leading to more accurate and efficient data collection, on the community level (24, 25). By connecting to DHIS2 via FHIR (Fast Healthcare Interoperability Resources) compliant APIs (Application Programming Interface), eCHIS automates the transfer of community level data into KHIS, reducing manual transcription delays and improving data completeness and timeliness (26). The eCHIS is a secure, interoperability program, that will be part of a broader Digital Health Platform in the future (21, 27). This system has significant implications for malaria control, as it enables Community Health Promoters (CHPs) to submit real-time malaria data, facilitating faster outbreak detection and response. Moreover, Kenya's partnership with Medic aims to “further strengthen eCHIS deployment, scaling it nationally” to improve malaria surveillance and intervention effectiveness (28), contributing to a cohesive, health information system.

Although eCHIS represents a promising advancement, its early testing phases revealed several limitations, including its dependence on sustained commitment from stakeholders and donors (24). During early digital health implementation efforts in Isiolo County (2019–2023) (29), CHPs reported that their responsibilities became increasingly demanding. The CHPs also mentioned operational issues like difficulty charging the phones and syncing data in areas that lack electricity and internet (29). Similar operational issues were also noted during the formal eCHIS pilot rollout in Migori County in 2022 (30), where approximately 1,500 CHPs were digitized with support from the Lwala Community Alliance and the CHU4UHC Platform (31). This can be particularly damaging since data quality may decrease, as CHPs may have to revert to paper-based reporting. This issue is particularly concerning for malaria surveillance, as delays in reporting may hinder rapid response efforts. The ongoing rollout of eCHIS aims to address these challenges, but as of now, both paper and electronic records are still commonly used in parallel. While Kenya has taken meaningful steps toward a unified digital health system, critical challenges remain in achieving full integration and efficiency. Ensuring that malaria-related data can be shared seamlessly across health facilities, CHPs, and research institutions requires not only interoperability but also investment in digital infrastructure, workforce training, and standardized reporting protocols. A fully integrated system will enable timely malaria case tracking, facilitate resource allocation, and enhance statistical analyses and predictions to prevent future outbreaks.

### 2.3 Data privacy and security

Although the Data Protection Act of 2019 enshrines the right to privacy and mandates lawful, fair, and transparent processing of personal and health data (32, 33), enforcement gaps exacerbated by delayed guidelines and limited regulatory oversight, have led to reported breaches, most notably during the COVID-19 pandemic when emergency surveillance measures compromised individual privacy rights (34). In response, the Office of the Data Protection Commissioner issued the Data Protection General Regulations in 2021, introducing requirements for data minimization, breach notification, and the registration of data controllers and processors, yet practical challenges in infrastructure, user training, and consistent enforcement persist (35). The Digital Health Act of 2023 further strengthened these safeguards by mandating that all digital health platforms explicitly comply with the Data Protection Act and by establishing a Digital Health Agency responsible for quality assurance, data governance, and national interoperability standards (36). This includes data security, a digital platform for the health sector, and proper data sharing to allow decision-making on any level (36). This act simultaneously addresses data privacy concerns, and regulation issues, and reiterates the need for a united digital health platform, with shareable data. The Act also creates a Digital Health Agency, whose main task is to “[establish and manage] an integrated health information system” (36). Nonetheless, the Finance Bill 2024s proposal to grant the Kenya Revenue Authority access to personal data without full adherence to the Data Protection Act has intensified debates over ethical governance and underscored the urgent need for robust, enforceable privacy frameworks that protect individual rights while enabling the rapid, secure data sharing essential for malaria surveillance and public health decision making (37).

### 2.4 Statistical analyses

Malaria modeling and surveillance in Kenya is a joint effort between the Kenyan government, Kenyan Institutions, and foreign entities. The Ministry of Health (MOH) tends to be at the center of these partnerships, with most reports coming directly from them. One of the main institutions performing statistical analyses on malaria is the Kenya National Bureau of Statistics (KNBS). The KNBS tends to report descriptive statistics on county demographics, malaria prevalence, and the efficiency of malaria prevention implementation. In the Kenya Demographic and Health Survey (KDHS), malaria is tracked nationally and county, among other community health factors (38, 39). A few metrics reported are percentages of homes with insecticide-treated nets (ITN) by endemicity zone and county, ITN usage, and reasons individuals may not use ITNs (38, 39). Since the KDHS reports on the overall health of the country, malaria is just a small portion of the report, with basic statistics recorded. In partnership with the MOH Division of National Malaria Program (DNMP), the KNBS completed an analysis for the Malaria Indicator Survey (MIS) as well. The MIS is a part of the international Roll Back Malaria initiative, with its final report dating 2020. This survey, like the KDHS, reported ITN usage and accessibility, malaria vaccinations, but also provided statistics on malaria prevalence by characteristics (i.e., child's age, sex, rurality, income, endemicity) and by malaria species (40). Through qualitative analysis, the MOH and KNBS can better recommend interventions to target individuals in high epidemic areas and keep track of trends. The MOH Division of National Malaria Program also provides reports of epidemiological and entomological factors, to monitor trends of malaria indicators. The Malaria Surveillance Bulletin (41), a quarterly report, records malaria prevalence and test positivity rates by region, seasonal transmission, and vector species composition. Like the MIS, this report tracks trends in malaria, with the most recent report being published for the April 2021–June 2021 quarter (41). One of the main objectives of the Kenya Malaria Strategy (KMS) is to strengthen malaria surveillance, ultimately leading to improved, evidence-based decision-making. With such a heavy emphasis on surveillance, the Kenyan government is laying a foundation to create a comprehensive malaria early warning system.

The Kenyan Meteorological Department, in partnership with the MOH and the Kenyan Medical Research Institute (KEMRI), frequently releases early warning predictions for malaria risk in the Kenyan highlands. These reports integrate climate variables such as maximum temperature, mean maximum temperature, temperature deviations, and rainfall into predictive models that estimate additive percentage risk (42). Comparing this additive risk to respective epidemic thresholds, the meteorological department and KEMRI assess the malaria epidemic risk in the upcoming months (42). These reports though, are limited to the Nandi, Kisii, and Kakamega districts (42), restricting their broader applicability.

To enhance malaria forecasting, researchers have explored advanced predictive models beyond Kenya. The Malaria Early Warning System (MEWS) integrates seasonal climate forecasts, routinely monitored climate data, and country vulnerability indices to predict malaria outbreaks (43). MEWS functions on a global scale, with reports covering multiple African countries, including Kenya. Additionally, machine learning approaches and Bayesian models have been increasingly utilized to predict malaria transmission patterns based on climate, entomological, and epidemiological data (44, 45). For instance, spatiotemporal models can identify hotspots of malaria transmission, while time-series models help in forecasting malaria case trends. While some research initiatives in Kenya incorporate predictive modeling, much of the MOH's malaria reporting remains surveillance based. Integrating predictive models into national reporting systems could significantly enhance emergency preparedness, optimize intervention strategies, and improve resource allocation for malaria control. Expanding early warning systems beyond the limited districts and incorporating machine learning-driven risk assessment tools could strengthen Kenya's malaria response framework, ultimately reducing disease burden and mortality (46, 47).

## 3 Discussion

**Figure 1:**
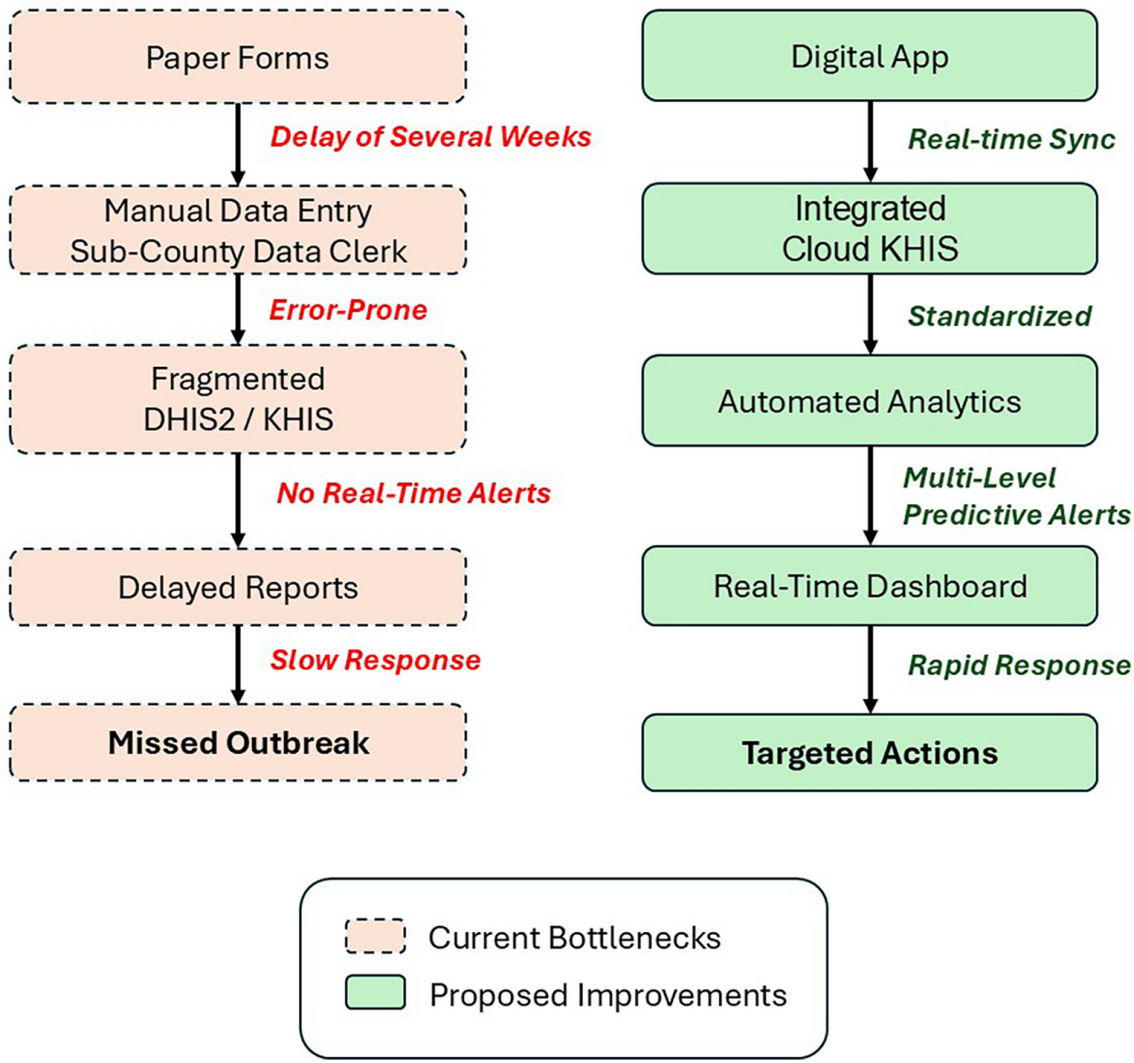
Comparison of malaria data flows in Kenya: current fragmented reporting vs. proposed integrated digital surveillance system. The left (red) side depicts Kenya's current system with paper-based delays and limited data, while the (green) shows proposed digital workflows enabling real-time analytics and targeted responses.

To effectively combat malaria in high-burden regions like Siaya County, Kenya, strengthening health information systems is essential for improving disease surveillance, resource allocation, and intervention strategies (8, 11–14). As illustrated in Figure 1 (left pathway), the current fragmented system, reliant mostly on paper-based forms and manual data entry, introduce significant delays in data reporting (48, 49), potentially extending up to several weeks. In addition, it limits variables to basic metrics (e.g., rapid detection test results), restricting spatial-temporal analyses and perpetuating reactive outbreak responses. This review highlights the need for an integrated (14), secured (33, 35, 37), interoperable (10, 15, 26), and data-informed approach to malaria control (22), leveraging real-time digital tools (48, 49) (e.g., mobile apps) and advanced statistical models (e.g., Bayesian spatiotemporal frameworks) to transform decision-making (13, 25, 28, 42–47).

The proposed digital workflow (Figure 1, right pathway) demonstrates how innovations at five critical levels from community data collection to national policy, can address systemic gaps such as: (1) Faster reporting: Mobile apps (9, 11, 30, 31, 48) reduce latency from weeks to hours by auto-syncing GPS-tagged case data and prevention metrics (e.g., use of insecticide-treated nets and indoor residual spraying), (2) Richer variables: Inclusion of mosquito species, breeding site proximity, and treatment adherence timestamps enables hotspot mapping and targeted interventions, (3) Automated analytics: Cloud-integrated dashboards that applies spatiotemporal models and machine learning techniques to improve monitoring and predictions (42, 46).

However, several limitations must be acknowledged. First, the proposed digital solutions assume stable electricity and internet connectivity, which remain inconsistent in rural malaria-endemic areas where less than half of the health facilities in western Kenya report reliable power access. Second, cultural and behavioral barriers among healthcare workers may hinder adoption, as observed in the eCHIS pilot (30, 31) where more than 30% of community health volunteers reverted to paper reporting due to fear of new technology or hesitancy on its use. Third, predictive models require high-quality historical data that may not exist in regions with chronically weak surveillance systems.

Key recommendations include the expansion of digital health platforms, improved data quality through standardized reporting mechanisms, and the incorporation of predictive modeling to identify high-risk areas and optimize intervention timing. These interventions should be coupled with: (a) solar-powered devices for offline data collection, (b) competency-based training programs adapted to local literacy levels (9, 17, 18), and (c) phased model deployment starting with data-rich districts. Investing in robust health information infrastructure will not only strengthen malaria control efforts in Kenya but also serve as a model for other malaria-endemic regions (2). A multi-sectoral approach, combining government agencies, healthcare providers, researchers, and community health workers, is necessary to bridge existing data gaps and ensure evidence-based policy decisions. Notably, our review is constrained by the limited evaluation period of digital interventions—most projects like eCHIS have operated for <2–3 years, making long-term sustainability assessments difficult. In addition, there is a need for foreign entities to collaborate with the local academic institutions, especially universities or research centers in sub-Saharan Africa. Subsequently, these local academic institutions can sustainably build the capacity of other stakeholders on the peripheral application of digital technology not only to control malaria but other infections. As the global health community continues to push for malaria elimination, this work highlights the critical role of data-informed strategies in transforming public health outcomes. Success of such initiatives could pave the way for broader applications, reinforcing the idea that sustainable disease control begins with reliable, accessible, and actionable data.

In conclusion, this review reveals that Kenya's malaria surveillance is hampered by fragmented data systems, manual reporting delays, and interoperability gaps, which undermine timely decision-making. Recent digital initiatives—DHIS2-based KHIS and the eCHIS rollout—have improved data capture and integration but still face infrastructure, training, and privacy challenges. Statistical analyses remain largely descriptive, with predictive modeling underutilized despite proven value in outbreak forecasting. Closing these gaps requires a unified digital platform that incorporates automated data flows, advanced analytics, and strong privacy safeguards. Such an integrated, data-driven approach will be pivotal for enhancing malaria control efforts in Western Kenya and beyond.
